# Prevalence of low back pain and associated occupational factors among Chinese coal miners

**DOI:** 10.1186/1471-2458-12-149

**Published:** 2012-03-01

**Authors:** Guangxing Xu, Dong Pang, Fengying Liu, Desheng Pei, Sheng Wang, Liping Li

**Affiliations:** 1 Injury Prevention Research Center, Medical College of Shantou University, 22 XinLing Road, Shantou 515041, China; 2 Shantou Center for Disease Control and Prevention, 58 Shanfen Road, Shantou 515041, China; 3 Institute of Health Research, University of Bedfordshire, Putteridge Bury, Luton LU2 8LE, UK; 4 Department of Occupational and Environmental Health, Peking University Health Science Center, 38 Xue Yuan Road, Beijing 100089, China

## Abstract

**Background:**

Very few studies have evaluated the association between occupational factors and low back pain (LBP) among miners. The epidemiological data on LBP in Chinese miners are limited. The aim of this study was to measure the prevalence of low back pain in Chinese coal miners and to investigate the role of occupational factors.

**Methods:**

A cross-sectional survey was conducted to examine 1573 coal miners in northern China. The prevalence of LBP over a 12-month period was assessed using the Nordic Musculoskeletal Questionnaire. Odds ratios were calculated to examine the association between the prevalence of LBP over a 12-month period and occupational factors using logistic regression.

**Results:**

Among the coal miners, 64.9% self-reported LBP in a 12-month period. Occupational factors associated with LBP were identified, including tasks with a high degree of repetitiveness (OR 1.3, 95%CI 1.0-1.6), tasks characterized by a high level of physical demand (OR 1.4, 95% CI 1.1-1.8), posture requiring extreme bending (OR 1.6, 95% CI 1.2-1.7) and insufficient recovery time (OR 1.4, 95% CI 1.0-1.8).

**Conclusion:**

Low back pain is common among Chinese miners. There were strong associations with occupational factors.

## Background

It is estimated that 37% of LBP is attributed to occupational factors, with twofold variation across regions [1]. Although LBP has been reported to be a common reason for absenteeism in the coal mining industry and for high health care costs [2-7], epidemiological data on LBP in Chinese miners are limited. One study showed a 12-month period prevalence of LBP to be 62.9% [8]. Interestingly, the study associated moisture, ventilation and trauma with LBP among these miners [8], which suggests a potential role of occupational factors in the aetiology of back pain. However, few studies have reported associations with other occupational factors. In this study, we adopted two internationally standardised questionnaires on occupational factors and LBP with the aim of providing insight to design preventive LBP interventions. Specifically, we assessed the prevalence of LBP among Chinese coal miners and investigated the role of detailed occupational factors using the Nordic Musculoskeletal Questionnaire (NMQ) [9] and the adapted Dutch Musculoskeletal Questionnaire (DMQ) [10].

## Methods

### Subjects

Nineteen hundred workers were cluster sampled from a coal-mining population in northern China and data collected from March 15-April 15, 2009. Underground miners and surface workers were included. Notably, all underground miners were male. After informed consent was obtained from each worker, the participants completed the set of questionnaires with the help of trained field epidemiologists. This research was approved by the Ethics Committee of the Medical College of Shantou University. The research was conducted in compliance with the Helsinki Declaration.

### Occupations

The underground miners performed a number of tasks, including a large degree of heavy weightlifting, bending, prolonged standing, and walking in a confined space. The surface workers' jobs involved transporting, servicing, and repairing equipment as well as administrative services at ground level.

### Low back pain

The 12-month period prevalence of LBP was assessed according to the Nordic Musculoskeletal Questionnaire. The NMQ has been documented to have acceptable validity and reliability. The common use of the questionnaire in epidemiological studies of LBP facilitated comparison of the findings between studies. The study participants were categorised according to whether they were "having or had pain or discomfort during the past 12 months." The subjects who answered "yes" were classified as having LBP. All others responding "no" were assigned to the non-LBP group.

### Personal and occupational factors

Personal and occupational factors were assessed using a self-administered questionnaire derived from the Dutch Musculoskeletal Questionnaire. Regarding personal factors associated with LBP, details were collected on age, gender, height, weight, working hours and education level as well as lifestyle factors, such as smoking. The DMQ was used to assess specific occupational factors, including information on lifting, standing, bending, twisting and vibrating, in addition to the types of jobs and tasks, work organisation and the main potential factors for LBP. Each questionnaire item included five alternative responses for each question (always, often, sometimes, seldom, and never/almost never). Work was categorised as tasks with a high degree of repetitiveness ( > 4 hours per day), performed with arms at or above shoulder level ( > 2 hours per day), insufficient recovery time ( < 10-minute back break), tasks characterized by a high level of high physical demand ( > 20 kg), posture characterized by extreme bending posture ( > 2 hours per day), etc. A pilot study of the test-retest reliability of the questionnaire was conducted to demonstrate that the questionnaires were reliable (Kappa 0.83).

### Statistical analysis

The differences between participants with and without back pain were examined by a Student's t-test (for continuous variables) and χ^2 ^test (for categorical variables). Odds ratios (ORs) and 95% confidence intervals (95%CI) were calculated to examine the association of LBP with occupational and personal factors using multiple logistic regression modeling. For logistic regression, the 12-month period prevalence of LBP used as the dependent variable. Independent variables were considered on the basis of previous epidemiologic and ergonomic studies. Initially, univariate analyses were calculated, with each of the potential explanatory variables as independent variables and LBP as the dependent variable. ORs were obtained for each potential factor after adjustment for age. In further multivariate logistic regression, non-significant variables (P > 0.05) were excluded, with the exception of age, which remained in the model regardless of statistical significance. Multivariate logistic regression analyses were performed using all retained variables. The final model included terms with a P value less than 0.05. All analyses were performed using SPSS version 15.0.

## Results

Of 1900 completed questionnaires, 1537 responses with more than 90% of the items completed were included in the analysis. The response rate was 80.9% among the participants. Participants' age was in the range of 19-61 years (35.2 ± 16.7 years). There were 1051 underground workers (68.4%) and 486 surface workers (31.6%).

The overall prevalence of back disorder over the preceding 12 months was 64.9% (n = 977). The prevalence of back disorder among underground workers was higher than among surface workers (67.2% vs 59.4%, *P *< 0.001). The miners with LBP were significantly older than those without LBP. The miners had been in service for a mean duration of 12.3 years (range: 0.5-41 years). The seniority of coal miners at their current job varied significantly between the groups. Education level was categorized into 1 of 4 groups: very low (5-6 years of school education), low (7-10 years), medium (11-14 years) and high (college education and higher). There were significant differences in education levels between the groups. A significant difference in working hours was found between the groups [Table 1].

**Table 1:** Characteristics of study subjects

Individual Characteristics	With LBP (N = 997)	Without LBP(N = 540)	*P*
Age(yrs)	36.8 ± 15.1, M = 37	32.3 ± 18.1, M = 40	< 0.001

Height(m)	1.71 ± 0.05	1.70 ± 0.06	0.45

Weight(kg)	71.1 ± 10.0	70.9 ± 10.0	0.53

BMI(kg/m2.)	24.0 ± 4.4	24.2 ± 3.8	0.22

Seniority in current job(yrs)	12.8 ± 10.1, M = 6	11.5 ± 10.2, M = 10	0.02

Working hours per week	52.1 ± 9.7, M = 48	49.9 ± 9.2, M = 50	0.007

Left-handed n(%)	135(13.5%)	71(13.1%)	0.69

Smoking n(%)	541(54.3%)	290(53.7%)	0.73

Education level n(%)			< 0.001

High	100(10.0%)	106(19.6%)	

Medium	206(20.7%)	117(21.7%)	

Low	636(63.8%)	294(54.4%)	

Very low	53(5.7%)	21(3.9%)	

M = median value

Univariate analyses [Table 2] showed a strong association between increasing age and LBP for all workers. A potential relationship between occupational factors and LBP was also identified for all workers. These results demonstrated that LBP was associated with the following factors: standing for long periods (OR 1.2, 95%CI 1.1-1.3), exposure to cold temperatures (OR 1.6, 95% CI 1.3-2.0), tasks characterized by a high degree of repetitiveness (OR 1.5, 95%CI 1.2-1.8), tasks requiring the arms to be at or above shoulder level (OR 1.5, 95%CI 1.3-2.0), tasks characterized by a high level of physical demand(OR1.8, 95%CI 1.4-2.2), posture characterized by extreme twisting(OR1.6, 95%CI 1.3-2.0), posture characterized by extreme bending(OR1.7, 95%CI 1.4-2.1) and static work posture (OR 1.4, 95%CI 1.1-1.7). There was a significantly higher proportion of complaints about having limited time for rest during a working day among those with LBP compared to those without LBP (OR2.0, 95%CI 1.6-2.7). Underground workers had a significantly higher risk of LBP than surface workers (OR1.4, 95% 1.2-1.8). Potential factors were examined simultaneously in multivariate logistic regression. LBP was associated with tasks characterized by a high degree of repetitiveness (OR 1.3, 95%CI 1.0-1.6), tasks characterized by a high level of physical demand (OR1.4, 95%CI 1.1-1.8), posture characterized by extreme bending (OR 1.4, 95%CI 1.0-1.7), and insufficient recovery time (OR 1.4, 95%CI 1.0-1.8). Separate analyses were carried out for underground miners and surface maintenance workers. Factors associated with LBP were a high degree of repetitiveness (OR 1.6, 95%CI 1.2-2.1), tasks characterized by a high level of physical demand (OR 1.6, 95% CI 1.2-2.0), posture requiring extreme bending (OR 1.4, 95% CI 1.1-1.9) and insufficient recovery time (OR 1.5, 95% CI 1.1-2.1) in underground miners (not shown). Factors associated with LBP were exposure to cold temperature(OR 2.0, 95%CI 1.4-2.9), tasks characterized by a high level of physical demand (OR 2.0, 95% CI 1.4-3.0), posture requiring extreme bending (OR 2.1, 95% CI 1.4-3.3) and insufficient recovery time (OR 2.4, 95% CI 1.6-3.5) in surface maintenance workers (not shown).

**Table 2:** Univariate and multivariate analyses for potential occupational factors of LBP in coal miners

	LBP		Univariate analysis(a)		Multivariate analysis(b)	
**Factors**	**No(n)**	**Yes(n)**	**OR(95%CI)**	***P***	**OR(95%CI)**	*P*

Age, years						

≤ 25(reference)	151	154				

26-35	88	169	1.9(1.3-2.7)	< 0.001	1.8(1.2-2.8)	0.009

36-45	140	276	2.4(1.8-3.2)	< 0.001	2.3(1.4-2.9)	< 0.001

> 46	160	392	1.9(1.4-2.6)	< 0.001	2.1(1.2-2.6)	0.007

Standing for long period						

No	218	270	1		1	

Yes	322	724	1.2(1.1-1.3)	< 0.001	1.0(0.8-1.3)	

Overtime works						

No	283	420	1		1	

Yes	257	577	1.5(1.2-1.9)	< 0.001	1.1(0.9-1.5)	

Exposure to cold temperature						

No	185	248	1		1	

Yes	349	740	1.6(1.3-2.0)	< 0.001	1.2(0.9-1.5)	

Tasks with a high degree of repetitiveness						

No	330	515	1		1	

Yes	202	466	1.5(1.2-1.8)	< 0.001	1.3(1.0-1.6)	0.034

Performed with arms at or above shoulder level						

No	325	505	1		1	

Yes	210	447	1.5(1.3-2.0)	0.001	0.9(0.7-1.3)	

Tasks characterized by a high level of physical demand ( > 20 kg)						

No	286	387	1		1	

Yes	249	599	1.8(1.4-2.2)	< 0.001	1.4(1.1-1.8)	0.011

Posture characterized by extreme bending						

No	248	313	1		1	

Yes	289	670	1.9(1.5-2.3)	< 0.001	1.4(1.0-1.7)	0.025

Posture characterized by extreme twisting						

No	289	407	1		1	

Yes	243	572	1.6(1.3-2.0)	< 0.001	1.1(0.8-1.4)	

Static work posture						

No	279	434	1		1	

Yes	253	550	1.4(1.1-1.7)	0.002	0.8(0.6-1.2)	

Insufficient recovery time						

No	199	238	1		1	

Yes	331	750	2.0(1.6-2.7)	< 0.001	1.4(1.0-1.8)	< 0.001

Type of work						

Surface	250	290	1		1	

Underground	290	707	1.4(1.2-1.8)	< 0.001	1.0(0.9-1.5)	

a 12 separate analyses adjusting for age

b Simultaneous analysis of all 12 variables with additional adjustment for age

## Discussion

This cross-sectional survey was conducted to investigate the prevalence of LBP in coal miners and the association of LBP with occupational and personal factors in miners.

### The 12-month period prevalence of LBP

The study demonstrated that the 12-month period prevalence of LBP was high in this study, affecting approximately 64.9% of Chinese coal miners. This finding is consistent with a report by Zejda et al., who found the 12-month period prevalences of 62.2% and 66.4% among coal miners at two mines. A recent study by Zhang et al. found that the prevalence of LBP among coal miners in China was 62.9% over 12 months [8,11]. Sarikaya reported that the prevalence of LBP over the past five years among underground and surface workers was 78% and 34%, respectively [12]. In Limburska's study, the 12-month period prevalence of LBP was 65.2% and 73.2% among coal miners at two mines [13]. It is reported that workers who perform heavy manual jobs have a higher prevalence of LBP and a higher level of absenteeism. Miners have the highest rate of absenteeism due to back pain [12]. This study supported previous reports that LBP is a common occupational health problem among Chinese miners. It is important to pinpoint such hazards to develop prevention strategies.

### Factors associated with LBP in coal miners

The causes of LBP in coal miners have not previously been described in detail. Few studies have been conducted on the relationship between occupational factors and LBP among coal miners.

### Personal factors

This study demonstrated that advancing age is strongly associated with LBP. In Hagberg's study, the normal degeneration of tissue with age predisposes individuals to LBP[14]. Our research is consistent with the epidemiology literature [15-19]. Notably, at present, it is difficult to distinguish between pathological degeneration and normal changes due to aging [20].

### Occupational factors

Epidemiological studies have demonstrated several combinations of physical factors related to LBP. The main physical factors causing musculoskeletal disorders include rapid work pace and repetitive motion patterns, insufficient recovery time, heavy lifting and other forceful manual exertions, non-neutral body postures (either dynamic or static) of the wrists, elbows, or shoulders, concentrations of mechanical pressure, vibration (both segmental and whole-body) and low temperature [1]. Among these factors, weightlifting and postural changes are the most significant factors causing low back pain among workers in heavy industry [12]. This finding highlights four work-related physical ergonomic factors used to categorise coal miners. This conclusion is in accordance with the epidemiology literature [12,17,18,21,22]. As reported in this study, underground miners have a higher risk of LBP than surface miners because of working-environment characteristics. The underground miners' jobs involve extensive lifting, bending, prolonged standing, and walking in a confined space [19]. The surface workers' jobs involve transporting, servicing, and repairing equipment as well as performing administrative duties at ground level. High physical demand was clearly associated with LBP, which was consistent with previous studies among coal miners [12,19]. The tasks performed by coal miners involve high-energy demands or require considerable physical strength. Numerous studies have been conducted on the relationship between excessive repetition and work-related musculoskeletal disorders [15,17,18,23]. Some previous studies have demonstrated a strong association between excessive repetition and LBP among the working population [21,24]. Our study further confirmed this finding among professional coal miners. In accordance with the epidemiological literature [16,19,21,24], extreme bending posture was highly associated with LBP. Among workers in heavy industry, postural changes are the most important risk occupational factor for LBP [12]. Previous studies revealed that working in a bent posture in confined spaces leads to higher intradiscal pressure and increased physical demand as compared to standing [23,25]. Among underground miners, extreme bending posture in a confined space was associated with LBP. Tasks requiring the arms to be at or above shoulder level represented a significant univariate factor for LBP, but no significant association remained when these factors were examined simultaneously. This may be due to the possible interaction between arms at or above shoulder level and high physical demand. In our study, insufficient recovery time over the course of a working day was strongly associated with LBP. The working times of professional coal miners far exceed those of the general working population; 70.9% of miners reported that they were required to work at least 50 hours each week.

### Methodological considerations

From a methodological perspective, this study has certain limitations. The sample comprised 80.9% of workers who were invited to fill out the questionnaires, including many retired workers. Therefore, there was a high participation rate. Subjects affected by LBP were more inclined to respond to the questionnaires than healthy subjects. The true prevalence would be expected to be lower than estimated by the present study.

Furthermore, because of the limited applicability of diagnostic procedures, the genuine "non-specificity" of pain states or both, epidemiological surveys may include a considerable proportion of cases without an identifiable pathophysiological basis [26]. Pain is a subjective experience, and there is no "gold standard" with which one may calibrate the instrument of measurement [27]. History of LBP was assessed according to the Nordic Musculoskeletal Questionnaire, which has been documented to have acceptable validity and reliability [28]. LBP was diagnosed based on the self-completion questionnaire; the incorporation of a physical examination would improve the quality of future studies.

Because the study had a cross-sectional design, cause-effect relationships regarding the observed associations cannot be established. However, the results of the study were suggestive of a possible relationship between personal and occupational factors and LBP. Because of the nature of this retrospective questionnaire survey, it is difficult to rule out the possibility of recall bias. In other words, respondents with complaints might recall exposure to work-related factors more accurately than respondents without complaints. For example, the workers with pain perceive exposures to be more extreme or adverse than workers without pain Thus, the true associations between the occupational factors examined and LBP may have been overestimated. Further prospective cohort studies would be helpful to confirm or refuse associations found in the present study.

## Conclusions

This study found a high prevalence (64.9%) of LBP over a 12-month period among Chinese coal miners, and certain personal and physical ergonomic factors were strongly associated with LBP. In particular, abnormal posture was highly associated with for LBP. Further prospective studies are required to confirm these findings. The study suggests that ergonomic studies will be of importance in identifying prevention strategies. LBP prevention strategies, such as the implementation of a break from work and reductions in workload, may be helpful.

## Competing interests

The authors declare that they have no competing interests.

## Authors' contributions

GX participated in the design of the study, conducted the statistical analysis, and drafted the manuscript. LL conceived the study, and took part in the interpretation of results. WS participated in the design of the study, FL and Ds P participated in the survey. DP helped to draft the manuscript. All the authors read and approved the final manuscript.

## Pre-publication history

The pre-publication history for this paper can be accessed here:

http://www.biomedcentral.com/1471-2458/12/149/prepub
